# Frequent Zika Virus Sexual Transmission and Prolonged Viral RNA Shedding in an Immunodeficient Mouse Model

**DOI:** 10.1016/j.celrep.2017.01.056

**Published:** 2017-02-14

**Authors:** Nisha K. Duggal, Jana M. Ritter, Samuel E. Pestorius, Sherif R. Zaki, Brent S. Davis, Gwong-Jen J. Chang, Richard A. Bowen, Aaron C. Brault

**Affiliations:** 1 Division of Vector-Borne Diseases, Centers for Disease Control and Prevention, Fort Collins, CO 80521, USA; 2 Division of High-Consequence Pathogens and Pathology, Centers for Disease Control and Prevention, Atlanta, GA 30333, USA; 3 Department of Biomedical Sciences, Colorado State University, Fort Collins, CO 80523, USA

## Abstract

Circulation of Zika virus (ZIKV) was first identified in the Western hemisphere in late 2014. Primarily transmitted through mosquito bite, ZIKV can also be transmitted through sex and from mother to fetus, and maternal ZIKV infection has been associated with fetal malformations. We assessed immunodeficient AG129 mice for their capacity to shed ZIKV in semen and to infect female mice via sexual transmission. Infectious virus was detected in semen between 7 and 21 days post-inoculation, and ZIKV RNA was detected in semen through 58 days post-inoculation. During mating, 73% of infected males transmitted ZIKV to uninfected females, and 50% of females became infected, with evidence of fetal infection in resulting pregnancies. Semen from vasectomized mice contained significantly lower levels of infectious virus, though sexual transmission still occurred. This model provides a platform for studying the kinetics of ZIKV sexual transmission and prolonged RNA shedding also observed in human semen.

## In Brief

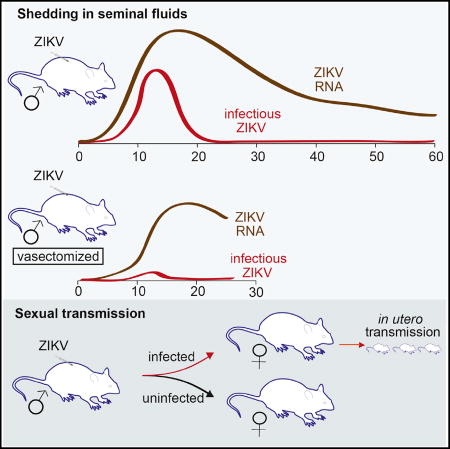

Duggal et al. describe a mouse model of ZIKV sexual transmission in which male mice shed ZIKV in semen and sexually transmit ZIKV to female mice. They demonstrate that viral RNA persists in semen for weeks after infectious virus is no longer detected in semen and that vasectomy significantly reduces infectious ZIKV in semen.

## INTRODUCTION

Zika virus (ZIKV; genus *Flavivirus* in the *Flaviviridae* family) is an emerging arbovirus that is primarily transmitted by *Aedes* spp. mosquitoes. Recently, ZIKV has established local mosquito-borne transmission across many parts of the Americas (Pan American Health Organization [PAHO], 2016). In addition to mosquito-borne and maternal-fetal transmission, sexual transmission of ZIKV in humans has been documented, initially, in a traveler returning to the United States from Africa (Foy et al., 2011), with subsequent isolation of ZIKV from the semen of an infected man during the ZIKV outbreak in French Polynesia in 2013 (Musso et al., 2015). Several cases of male-to-female (Hills et al., 2016), a case of male-to-male (Deckard et al., 2016), and a suspected case of female-to-male (Davidson et al., 2016) sexual transmission of ZIKV have been reported in travelers returning to the United States from regions affected by the ongoing outbreak in the Americas

In infected males, infectious virus has been isolated from semen up to 24 days after the onset of symptoms (D’Ortenzio et al., 2016), and perhaps longer, although the culture methods of many reports are unclear. ZIKV RNA has been detected in semen up to 6 months post-symptom onset (Barzon et al., 2016; Nicastri et al., 2016) and in the semen of a vasectomized man up to 96 days post-symptom onset (Arsuaga et al., 2016); however, the infectivity and transmission potential of persistent ZIKV RNA in semen is not known. Of significant concern, a case of male-to-female sexual transmission of ZIKV from an asymptomatic male traveler to a woman with no travel history has been reported (Brooks et al., 2016). This finding could indicate that transmission through seminal shedding is possible with minimal or no symptoms. Given the severe disease that ZIKV can cause in developing fetuses (Rasmussen et al., 2016), the risk of sexual transmission to women during pregnancy is of particular concern.

Recently developed mouse models of ZIKV pathogenesis have shown that genetically immunodeficient mice develop neurological disease in adults and fetal demise in pregnant females (Lazear et al., 2016; Miner et al., 2016; Rossi et al., 2016; Yockey et al., 2016), whereas immunocompetent mice are not susceptible to ZIKV peripheral inoculation (Lazear et al., 2016; Rossi et al., 2016). In addition, a non-human primate model of ZIKV pathogenesis has shown brain lesions in the fetuses of inoculated pregnant macaques (Adams Waldorf et al., 2016) and ZIKV RNA in the seminal fluids of inoculated male macaques at 7 and 14 days post-inoculation (Osuna et al., 2016). These studies have demonstrated the presence of infectious ZIKV in many tissues, including testes and placenta, of adults during acute infection and in the fetuses of pregnant females. Persistence of viral RNA from a mouse-adapted strain of ZIKV in the testes of inoculated mice has been shown through day 21 (Govero et al., 2016). However, the potential for sexual transmission of ZIKV and the relationship between viral RNA and infectious virus in semen have not been directly assessed in an animal model.

In this study, we evaluated the pathogenesis and sexual transmission of a contemporary ZIKV strain (PRVABC59; Puerto Rico 2015) (Lanciotti et al., 2016) in interferon α/β and -γ receptor knockout AG129 mice. Testes of inoculated male mice were persistently infected with ZIKV for more than 4 weeks, and semen contained infectious virus from days 7 through 21 post-inoculation. Semen from inoculated male mice contained ZIKV RNA for 5 weeks after infectious virus was no longer detectable. However, ZIKV titers in seminal fluids were reduced significantly when male AG129 mice were vasectomized prior to inoculation. In addition, ZIKV infection was documented in 50% of female AG129 mice that were mated to infected non-vasectomized male AG129 mice during days 7 through 19 post-inoculation, demonstrating a high frequency of sexual transmission in this model.

## RESULTS

### Experimental AG129 Mouse Model of Sexual Transmission of ZIKV

**Figure 1:**
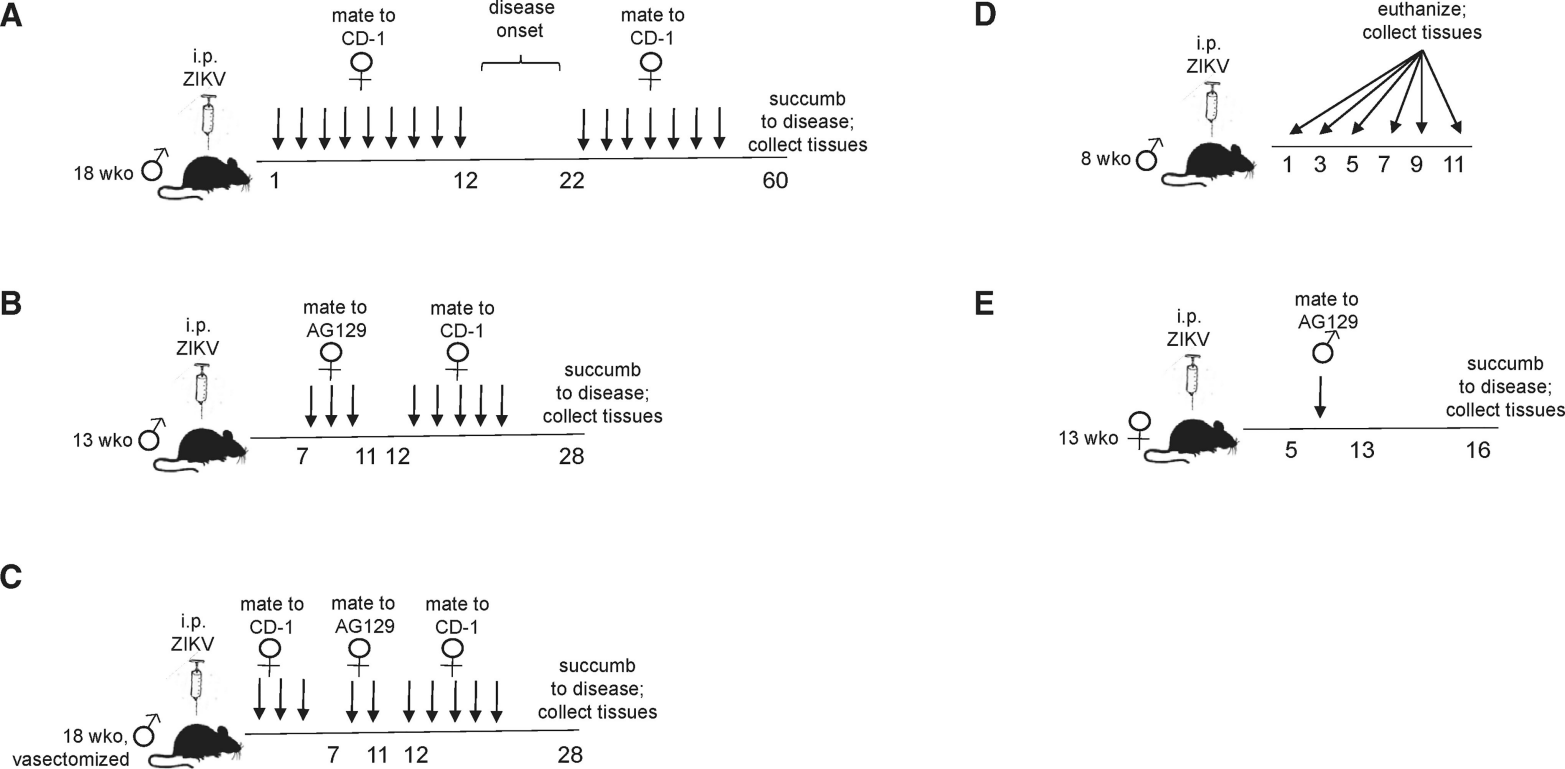
Experimental Design (A) Fourteen 18-week-old (wko) male AG129 mice were inoculated i.p. and mated to female CD-1 mice daily to collect seminal fluids until the male mice were euthanized due to disease progression. (B) Eleven 13-week-old male AG129 mice were inoculated i.p., mated to female AG129 mice for sexual transmission studies, and then mated to female CD-1 mice daily to collect seminal fluids until the male mice were euthanized. (C) Eight 18-week-old AG129 male mice were vasectomized before intraperitoneal inoculation and then mated to female CD-1 and AG129 mice to collect seminal fluids and assess sexual transmission until the male mice were euthanized. (D) Eighteen 8-week-old male AG129 mice were inoculated i.p. and euthanized in groups of three at prescribed days 1, 3, 5, 7, 9, and 11 post-inoculation. (E) Eight 13-week-old female AG129 mice were inoculated i.p. and mated to male AG129 mice for sexual transmission studies.

We used a combination of interferon α/β and -γ receptor knockout AG129 mice and outbred CD-1 mice (used as surrogate females for collecting semen) in five separate experiments. In order to assess the pathogenesis of PRVABC59, 18-week-old male AG129 mice (vasectomized and non-vasectomized) and 13-week-old male AG129 mice were inoculated intraperitoneally (i.p.) and followed for disease progression (Figures 1A–1C). These ages were chosen to maximize mating activity and because age may be associated with ZIKV shedding in semen in humans (unpublished data). To assess the tissue distribution of ZIKV, we collected tissues from symptomatic male mice and also from 8-week-old male AG129 mice that were inoculated i.p. and euthanized in groups of three on days 1, 3, 5, 7, 9, and 11 post-inoculation (Figures 1A–1D). To determine the kinetics of ZIKV shedding in semen, infected vasectomized or non-vasectomized male AG129 mice were mated to uninfected female surrogate CD-1 mice, and seminal fluids were collected from the uteri of mated CD-1 females (Figures 1A–1C). To determine the rate of male-to-female sexual transmission, infected non-vasectomized and vasectomized male AG129 mice were mated to uninfected female AG129 mice (Figures 1B and 1C), and the mated females were followed for disease progression. Finally, to assess the possibility of female-to-male sexual transmission, eight infected 13-week-old female AG129 mice were mated to uninfected male AG129 mice, and the mated male AG129 mice were followed for disease progression (Figure 1E).

### Persistence of Infectious ZIKV in the Testes of Male AG129 Mice

**Figure 2:**
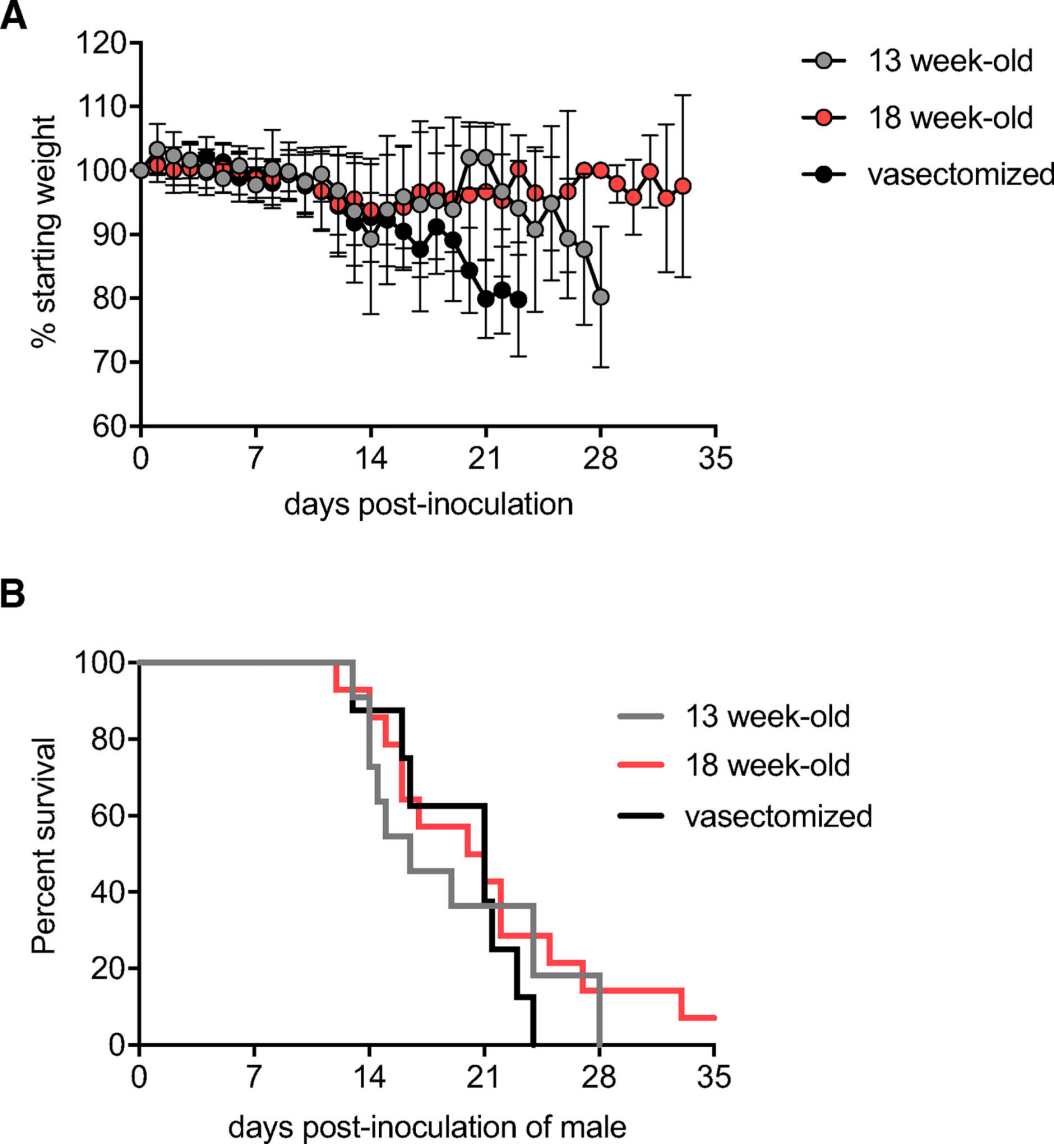
Pathogenesis of ZIKV Strain PRVABC59 in AG129 Mice (A) Average weight of mice post-inoculation, represented as a percentage of initial weight. Gray circles represent 13-week-old mice (n = 11), red circles represent 18-week-old mice (n = 14), and black circles represent vasectomized 18-week-old mice (n = 8). Error bars represent SD. (B) Percent survival of mice post-inoculation. Gray line represents 13-week-old mice, red line represents 18-week-old mice, and black line represents vasectomized 18-week-old mice.

Fourteen 18-week-old male AG129 mice, eight vasectomized 18 week-old mice, and eleven 13-week-old AG129 mice were inoculated i.p. with ZIKV strain PRVABC59. Weight loss and neurological signs were monitored daily. Mice were euthanized when they demonstrated definitive signs of encephalitis (e.g., incoordination, limb paralysis or weakness, recumbency) or conjunctivitis precluding vision. The first signs of disease were observed at 10 days post-inoculation, and weight loss was associated with the onset of observed symptoms, with greater weight loss seen in the vasectomized males compared to non-vasectomized males (p < 0.05). Survival was similar between the three groups of mice (Figure 2A, p = 0.66). While the majority of inoculated mice were euthanized due to disease progression within 28 days post-inoculation, 14% of non-vasectomized 18-week-old mice were maintained 4–5 weeks post-inoculation, and one non-vasectomized 18-week-old mouse remained healthy through week 8, at which point the study was terminated (Figure 2B). Tissues were collected at the time of euthanasia and tested for infectious ZIKV (described later).

**Figure 3:**
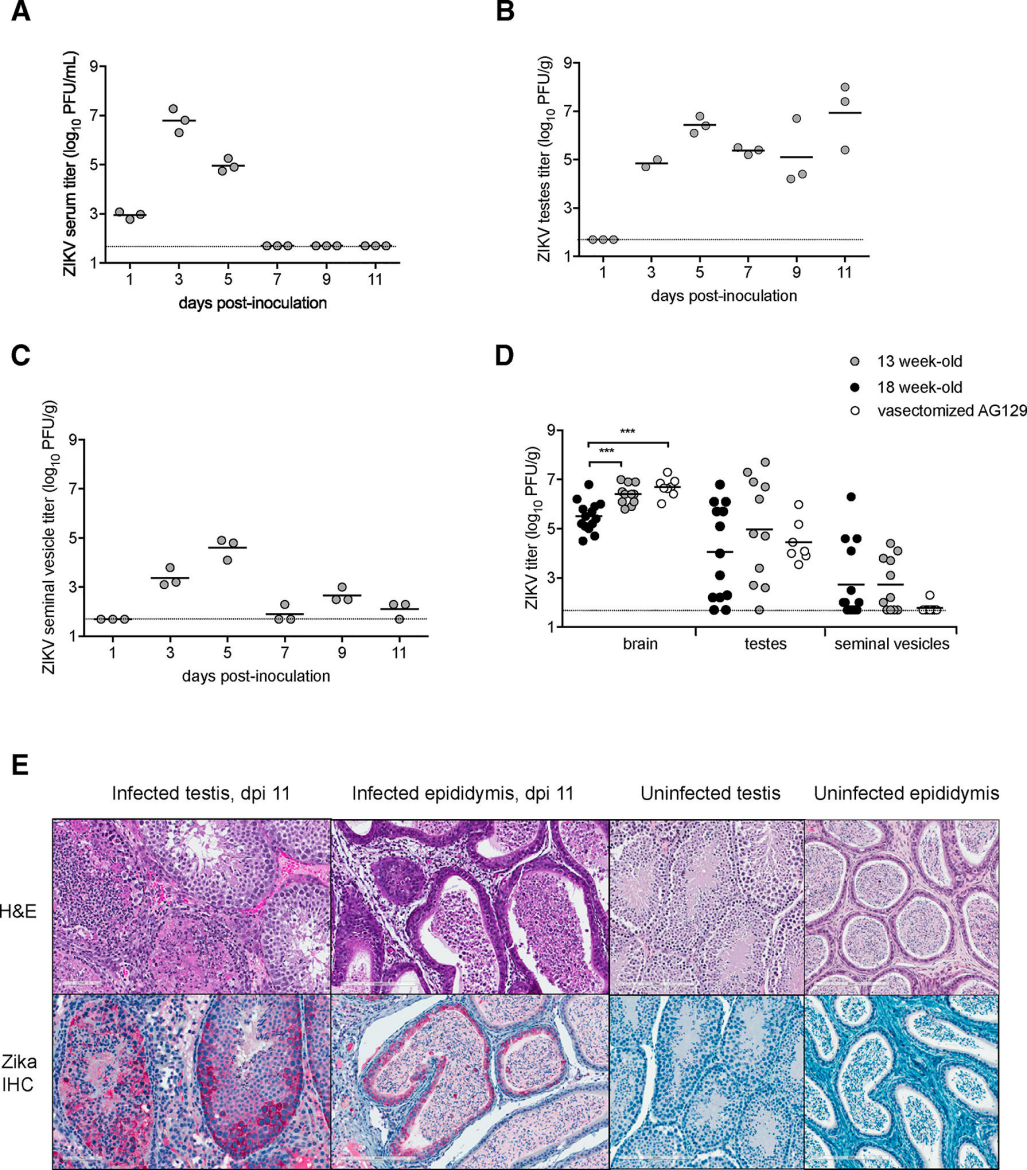
Infectious ZIKV in Male AG129 Mice Titers of samples from individual mice are represented by circles, with means represented by solid lines. The limit of detection (1.7 log_10_ PFU/mL or PFU/g) is represented by a gray line. (A) ZIKV titer in serum of 8-week-old mice (n = 15). (B) ZIKV titer in testes/epididymides of 8-week-old mice (n = 15). (C) ZIKV titer in seminal vesicles of 8-week-old mice (n = 15). (D) ZIKV titer in tissues collected from 18-week-old mice (n = 14), 13-week-old mice (n = 11), and vasectomized mice (n = 8) between days 12 and 59 post-inoculation. ***p < 0.001. (E) Inflammation and immunohistochemical localization of ZIKV in AG129 testis and epididymis. Top left: severe interstitial and seminiferous tubule inflammation with germ cell degeneration and sloughing on day 11; H&E, 200×. Bottom left: immunohistochemical localization of ZIKV in spermatogenic precursors in an inflamed region of testis on day 11; 400×. Top middle: interstitial inflammation and epididymal tubular dilation by cellular debris (sloughed germ cells) on day 11; H&E, 200×. Bottom middle: immunohistochemical localization of ZIKV in epididymal tubular epithelia and luminal cell debris on day 11; 400×. Top right: lack of inflammatory and degenerative changes in uninfected testis; H&E, 200×. Bottom right: lack of immunohistochemical staining in uninfected testis; 400×. Far right, top: lack of inflammation and tubular dilation in uninfected epididymis; H&E, 200×. Far right, bottom: lack of immunohistochemical staining in uninfected epididymis; 400×.

In order to study the acute kinetics of ZIKV infection, eighteen 8-week-old male AG129 mice were inoculated i.p., and three mice were euthanized every other day from days 1 to 11 post-inoculation. The infectious ZIKV titer in serum, brain, testes, and seminal vesicles was measured by plaque assay from tissues of 8-, 13- and 18-week-old mice. During acute ZIKV infection, mean serum titers peaked on day 3 post-inoculation at 6.8 log_10_ PFU (plaque-forming units)/mL (Figure 3A). Infectious virus was disseminated to testes/epididymides and seminal vesicles by 3 days post-inoculation (Figures 3B and 3C) and persisted in testes/epididymides through day 33 and in seminal vesicles through day 21. Dissemination of virus to testes and seminal vesicles was slightly altered in vasectomized mice compared to non-vasectomized mice of the same age, with a mean brain titer of 6.7 log_10_ PFU/g and 5.5 log_10_ PFU/g of tissue, respectively (Figure 3D, p < 0.001); a mean testes titer of 4.4 log_10_ PFU/g and 4.0 log_10_ PFU/g of tissue, respectively (Figure 3D, p = 0.61); and a mean seminal vesicle titer of 1.8 log_10_ PFU/g and 2.7 log_10_ PFU/g of tissue, respectively (Figure 3D, p = 0.09).

### Cellular Localization of ZIKV within the Testes of Male AG129 Mice

To investigate the cellular localization of ZIKV in the reproductive tract of male AG129 mice, histopathologic evaluation with immunohistochemistry for ZIKV was performed on the tissues collected from the 8-week-old mice. Acute inflammation and mixed inflammation were observed in the epididymides and testes, beginning at 3 and 5 days post-inoculation, respectively, with increasing occurrence and severity through 11 days post-inoculation, when all three mice from this time point had prominent inflammatory infiltrates involving the seminiferous and epididymal tubules, accompanied by variable degrees of germ-cell degeneration and sloughing compared to negative controls (Figure 3E). Beginning at 9 days post-inoculation, immunostaining of ZIKV antigens was consistently seen in testes and epididymides that had inflammation but was not always directly associated with inflammatory foci. Immunostaining localized to spermatogenic precursors, epididymal epithelia, and lumenal cell debris, as well as, to a much lesser extent, inflammatory cells. The seminal vesicle from only one mouse on day 11 post-inoculation showed focal epithelial staining.

### ZIKV RNA Persistence in Seminal Fluids of Male AG129 Mice

**Figure 4:**
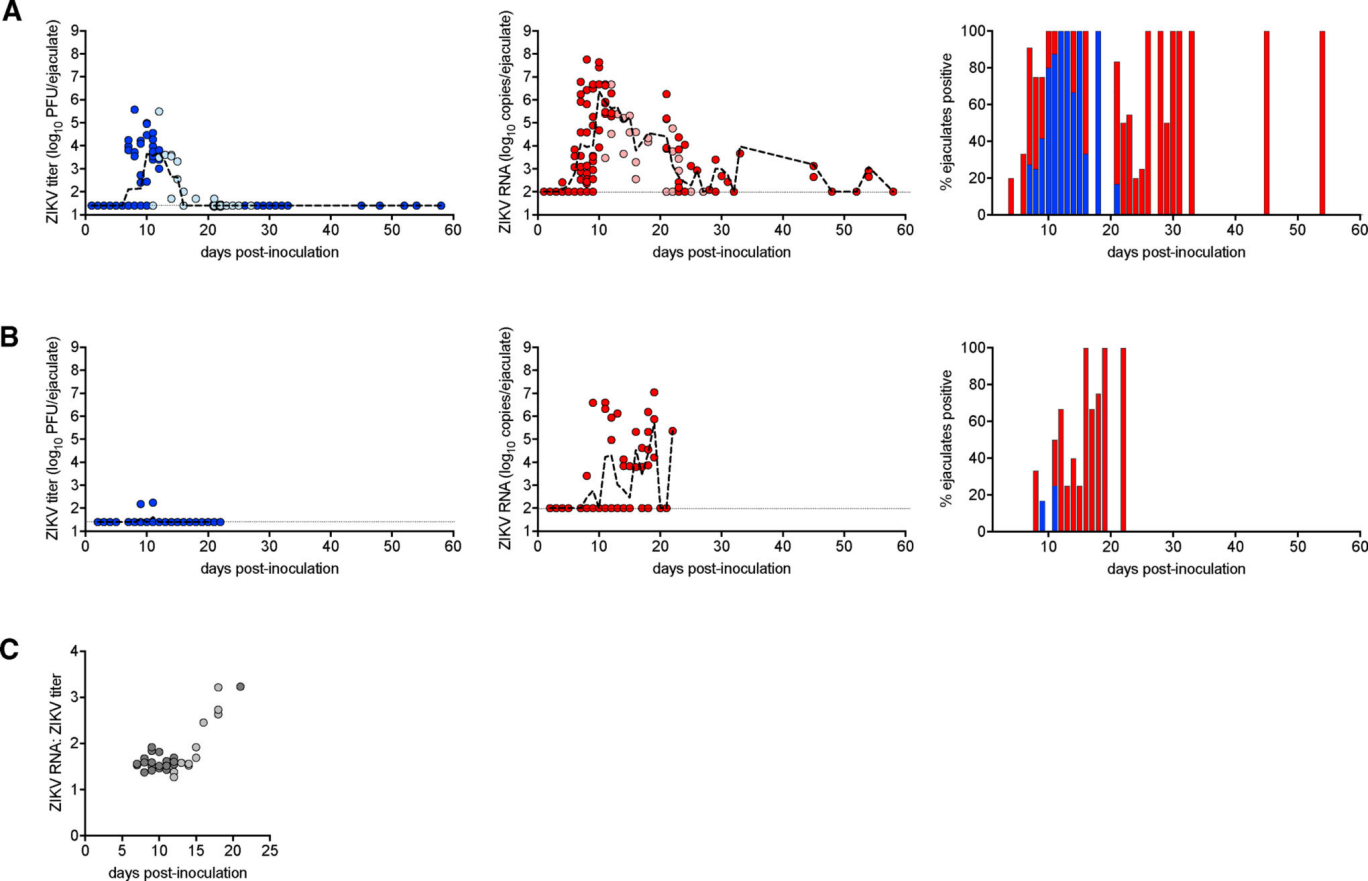
Shedding of ZIKV Infectious Virus and Viral RNA in Seminal Fluids of Male AG129 Mice Measurements of individual mating events (n = 217) occurring on days 1 through 58 post-inoculation are represented by circles. Light circles are samples from 13-week-old mice (n = 33), and dark circles are samples from 18-week-old mice (n = 116) or vasectomized 18-week-old mice (n = 68). Dashed lines represent mean values across all mice and are used to make trends more visible. (A) Non-vasectomized males. Left panel: ZIKV titer in seminal fluids per ejaculation post-inoculation. Middle panel: ZIKV RNA copy number in seminal fluids per ejaculation post-inoculation. Right panel: percentage of ejaculates obtained from mated CD-1 females containing ZIKV or viral RNA as a function of time post-male inoculation. (B) Vasectomized males. Left panel: ZIKV titer in seminal fluids per ejaculation post-inoculation. Middle panel: ZIKV RNA copy number in seminal fluids per ejaculation post-inoculation. Right panel: percentage of ejaculates obtained from mated CD-1 females containing ZIKV or viral RNA as a function of time post-male inoculation. (C) Ratio of ZIKV RNA copy number to ZIKV titer in individual ejaculations over time.

The 18-week-old AG129 mice were also used to study ZIKV persistence in semen. Seminal fluids were collected from the uteri of 116 female CD-1 mice that were mated to male AG129 mice that had been inoculated between 1 and 58 days prior to mating. Infectious ZIKV titers and viral RNA copy numbers were determined for each sample. Infectious ZIKV was detected in the ejaculates of AG129 mice between days 7 and 21 post-inoculation, with a mean peak titer of 3.8 log_10_ PFU per ejaculate on day 9 post-inoculation (Figure 4A, first panel). The sample with the highest viral titer contained 5.6 log_10_ PFU per ejaculate on day 8 post-inoculation. In contrast, viral RNA was detected in the ejaculates of male AG129 mice from day 4 through 58 post-inoculation, with a mean peak copy number of 6.1 log_10_ copies per ejaculate on day 10 post-inoculation (Figure 4A, second panel).

A second set of seminal fluid collections was performed from the uteri of 33 female CD-1 mice mated to 13-week-old AG129 mice that had been inoculated between 11 and 27 days prior to mating. A similar profile of infectious virus and viral RNA was observed between this group and the 18-week-old AG129 mice described earlier (p = 0.68 and p = 0.69, respectively). Infectivity of seminal fluids peaked on day 12 post-inoculation, with a mean titer of 4.2 log_10_ PFU per ejaculate (Figure 4A, first panel), and viral RNA peaked on day 12 with a mean peak copy number of 5.6 log_10_ copies per ejaculate (Figure 4A, second panel). Infectious virus was detected through day 21 post-inoculation, and viral RNA was detected in seminal fluid through day 23 post-inoculation. The semen sample with the highest viral titer in this group of mice contained 5.2 log_10_ PFU per ejaculate on day 12 post-inoculation.

In both studies, the ratio of viral RNA copy number to infectious particles followed a consistent, linear relationship from day 7 through day 15 post-inoculation (Figure 4C). However, the ratio of RNA to virus began to increase on day 16 post-inoculation, suggesting the onset of a host immune response that reduced infectivity of viral particles.

To evaluate the contribution of the testes to ZIKV shedding in semen, seminal fluids were collected from the uteri of 68 CD-1 females that were mated to vasectomized AG129 mice that had been inoculated between 1 and 23 days prior to mating. The seminal fluids from vasectomized males had significantly lower ZIKV titers compared to non-vasectomized males (p < 0.001) and a short interval of infectivity. Infectious ZIKV was detected from days 9 through 11 post-inoculation, with a mean peak titer of 1.6 log_10_ PFU per ejaculate on day 11. The sample with the highest viral titer contained 2.2 log_10_ PFU per ejaculate on day 9 post-inoculation (Figure 4B, first panel). However, the seminal fluids from vasectomized males contained similar levels of viral RNA compared to non-vasectomized males, with a mean peak copy number of 5.7 log_10_ copies per ejaculate on day 19 post-inoculation (Figure 4B, second panel, p = 0.24), suggesting that prolonged ZIKV RNA shedding in semen may not be derived from the same source as infectious virus.

### Intravaginal Inoculation of Female AG129 Mice

**Figure 5:**
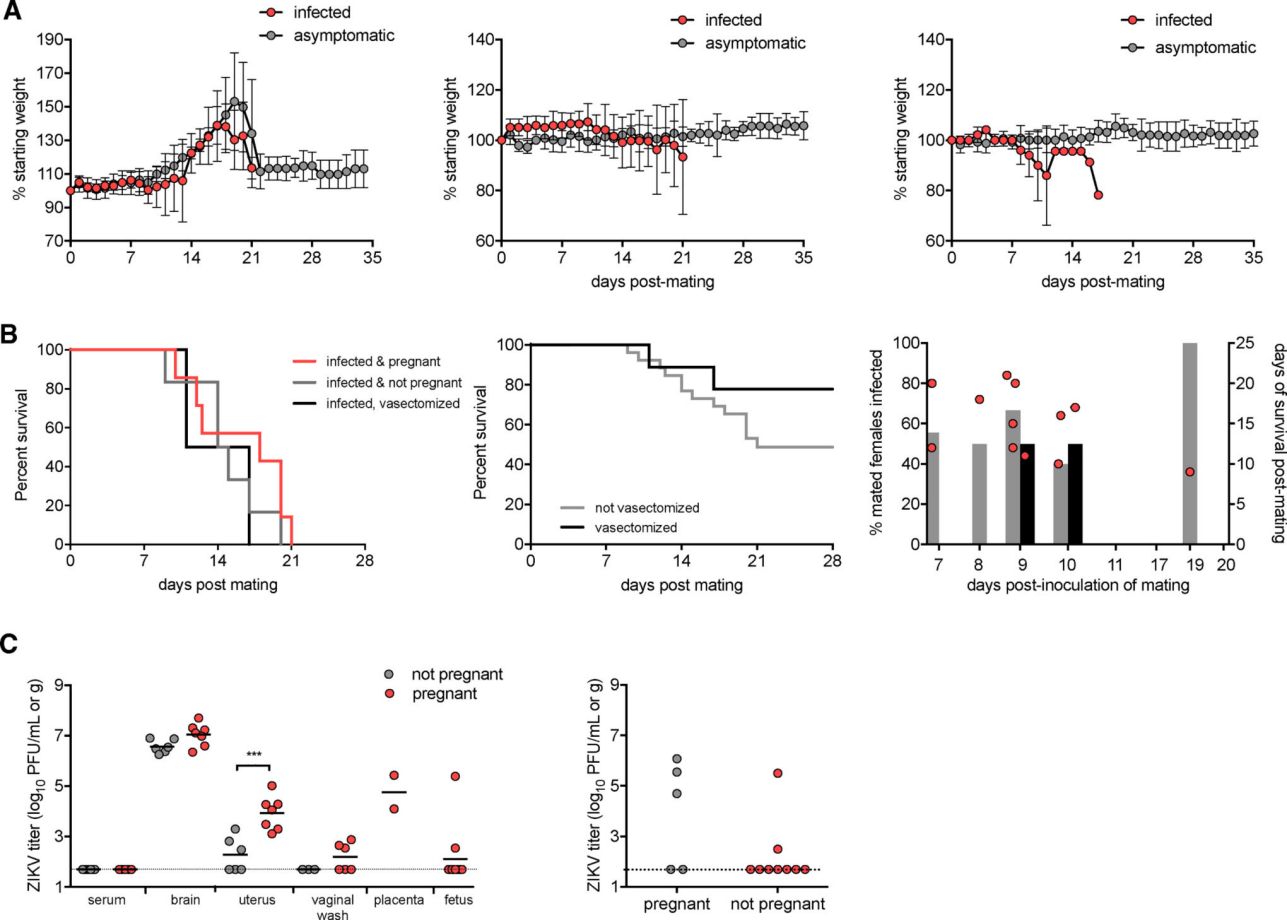
Male-to-Female ZIKV Sexual Transmission (A) Average weight of pregnant female mice (left panel; n = 13), non-pregnant female mice post-mating (middle panel; n = 13), and female mice mated to vasectomized males (right panel; n = 9) represented as a percentage of initial weight. Error bars represent SD. (B) Left panel: percent survival of infected pregnant female mice, infected non-pregnant female mice, and infected female mice mated to vasectomized males post-mating. Middle panel: percent survival of females mated to vasectomized (dark line) or non-vasectomized (light line) males. Right panel: percent of mated females that became infected on each day post-inoculation of male partner. Gray bars represent females mated to non-vasectomized males, and dark bars represent females mated to vasectomized males. Circles represent the days of survival post-mating of individual infected female mice. (C) Left panel: ZIKV titer in serum, brain tissue, uterus, vaginal wash, placenta, and fetus of sexually infected females collected on days 10–21 post-inoculation (n = 13). Gray circles represent non-pregnant infected females (n = 6), and red circles represent pregnant infected females (n = 7). Right panel: virus isolated from intravaginally inoculated pregnant (n = 5) and non-pregnant (n = 9) female mice. Titers are log_10_ PFU/g for brain and uterus tissue and log_10_ PFU/mL for serum and vaginal washes. The limit of detection (1.7 log_10_ PFU/mL or PFU/g) is represented by a dashed line. Individual mice are represented by circles, with means represented by lines. ***p < 0.001.

In order to assess the susceptibility of AG129 females to intravaginal infection, five pregnant and nine non-pregnant AG129 females were inoculated with ZIKV intravaginally (i.vg.). The pregnant mice were i.vg. inoculated between 6 and 10 days of gestation, and the non-pregnant mice were inoculated i.vg. at random stages of their cycle. Given the timing, during gestation, of inoculation, euthanization occurred prior to the exhibition of neurological signs; therefore, serum viremia was used to detect infection in these mice. Non-pregnant females were euthanized between 5 and 9 days post-inoculation, and serum and brain tissue were used to detect infection. Of pregnant females inoculated with ZIKV, 60% had virus in serum, and of non-pregnant females inoculated with ZIKV, 22% were infected, as determined by isolation of virus in serum or brain tissue (Figure 5C, second panel; p = 0.27). Infection i.vg. clearly showed a potential for male-to-female sexual transmission of ZIKV.

### Sexual Transmission of ZIKV in AG129 Mice

The aforementioned eleven 13-week-old male AG129 mice that were inoculated i.p. with ZIKV and bred to CD-1 females on days 11 through 27 post-inoculation were initially mated to 26 uninfected female AG129 mice, beginning at day 7 post-inoculation. This timing for mating to female AG129 mice was chosen because day 7 was the first time point post-inoculation when infectious virus was isolated from the seminal fluids of inoculated males (Figure 4). Females were checked each morning for the presence of a copulatory plug as evidence of mating, and mated females were weighed daily and followed for symptoms of disease. The majority of females were mated to males between 7 and 10 days post-inoculation, with one female mated each on day 11 and on day 19 post-inoculation. Body weights increased for the 13 out of 26 (50%) mated female mice that became pregnant, followed by weight loss after birthing (Figure 5A, first panel). Disease-associated weight loss was observed for infected females, compared to asymptomatic females with the same pregnancy status.

Eight out of eleven (73%; 95% confidence interval [CI]: 43%–91%) inoculated male AG129 mice transmitted ZIKV to at least one female AG129 mouse. Of 26 mated females, 13 (50%; 95% CI: 32%–68%) succumbed to infection, with no statistically significant differences between the survival of infected pregnant and infected non-pregnant females (Figure 5B, first panel, p = 0.22). Seven of the 13 pregnant females (54%) became infected, and 6 of the 13 non-pregnant females (46%) became infected. The percentage of mated females that became infected was consistently near 50% on mating days 7 through 10 (Figure 5B, third panel). The female mated on day 11 post-inoculation did not become infected, but the female mated on day 19 post-inoculation did become infected, which was supported by the identification of infectious virus in semen through day 21 post-inoculation, as described earlier.

The negative infection status of healthy females was confirmed by failure to detect neutralizing antibody by PRNT_90_ in any of the 13 surviving females at days 30–36 post-mating. The infection status of symptomatic females was confirmed by isolation of virus from brain tissue, which contained a mean titer of 6.8 log_10_ PFU/g of tissue (Figure 5C, first panel). None of the symptomatic females succumbed to infection during the acute phase when viremia was detectable in the time-course assayed males, and all serum samples obtained from these females at the time of euthanasia were negative for infectious virus (Figure 5C, first panel). Two of the seven pregnant infected females, which were mated on day 7 post-inoculation of the male, had evidence of fetal demise at days 10 and 12 post-mating, respectively. Interestingly, virus was also isolated from the uteri from 10 of 13 infected females with a mean titer of 3.6 log_10_ PFU/g of tissue (Figure 5C, first panel) and from the vaginal wash from 4 of 9 infected females with a mean titer of 2.4 log_10_ PFU per wash (Figure 5C, first panel), suggesting the potential for female-to-male sexual transmission of ZIKV. In utero transmission of ZIKV was evident in 2 out of 11 fetuses, both from one infected female that was mated on day 9 post-inoculation of the male (Figure 5C, first panel). A higher mean titer was evident in uteri from pregnant females (3.9 log_10_ PFU/g) compared to non-pregnant females (2.3 log_10_ PFU/g) (p < 0.001), further supporting the potential that pregnancy status may affect ZIKV susceptibility, as suggested in the intravaginal challenge of pregnant and non-pregnant AG129 females.

The eight vasectomized male AG129 mice that were inoculated i.p. were mated to 11 uninfected female AG129 mice beginning at day 7 post-inoculation. Two out of nine (22%; 95% CI: 5%–56%) mated females became infected, and the survival of these females was not statistically different than the survival of females mated to non-vasectomized male AG129 mice (Figure 5B, second panel, p = 0.19). The negative infection status of healthy females was confirmed by the failure to detect neutralizing antibody with a plaque-reduction neutralization test (PRNT) using a 90% cutoff value (PRNT_90_) in any of the seven surviving females at days 28–36 post-mating. The infection status of symptomatic females was confirmed by the isolation of virus from brain tissue. Interestingly, the two infected females were mated to the two vasectomized males from which infectious virus was detected in seminal fluids, suggesting that low levels of infectious virus in semen are sufficient for sexual transmission and that testes are not the only source of virus leading to sexual transmission.

**Figure 6:**
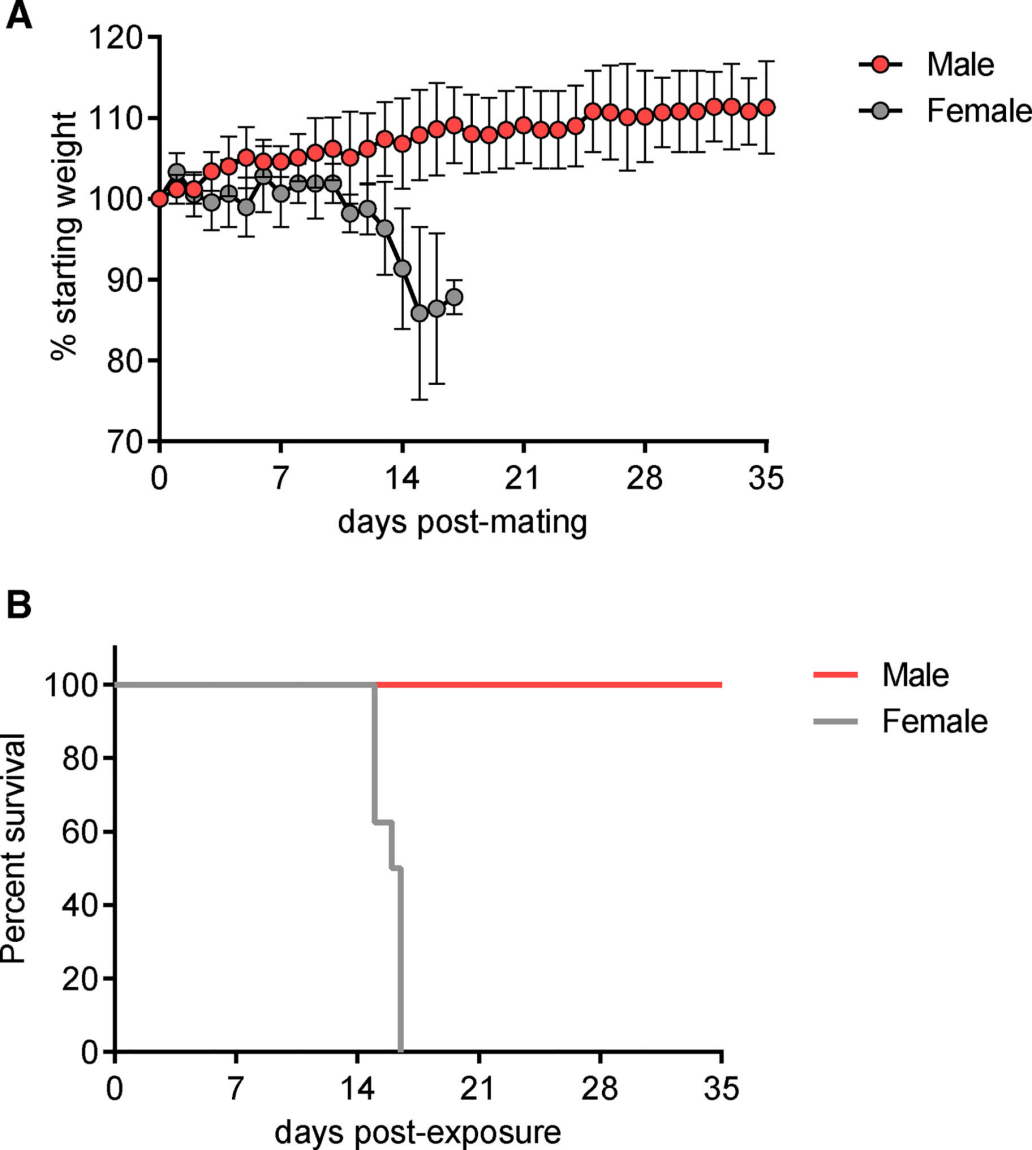
Female-to-Male ZIKV Sexual Transmission (A) Average weight of female mice (n = 8) post-inoculation and average weight of male AG129 mice (n = 6) post-mating, represented as a percentage of initial weight. Error bars represent SD. (B) Percent survival of female mice post-inoculation and percent survival of male mice post-mating.

In order to explore the possibility of female-to-male sexual transmission of ZIKV, eight female AG129 mice were inoculated i.p. with ZIKV and exposed individually to uninfected male AG129 mice on days 3 (n = 3), 6 (n = 3), and 9 (n = 2) post-inoculation. Six matings were observed on days 5 through 13 post-inoculation of mated females. Inoculated females showed significant weight loss and 100% mortality by day 17 post-inoculation (Figures 6A and 6B). Mated male mice were weighed daily and were followed for signs of disease. No signs of disease or weight loss were observed in the male mice through day 30 post-mating (Figures 6A and 6B), and the lack of infection in males was confirmed by the failure to detect neutralizing antibodies in serum by PRNT. Infection of females was confirmed by positive plaque assay of virus from brain tissue. Additionally, sera, vaginal washes, and uteri were screened for infectious virus by plaque assay. Interestingly, ZIKV was not isolated from the uterus or vaginal wash of these i.p. inoculated females, suggesting a possible difference in viral dissemination in females, depending on the route of inoculation (sexual transmission versus i.p.).

## DISCUSSION

Seminal fluids of male AG129 mice were observed to contain infectious ZIKV beginning at 7 days post-inoculation and continued through 21 days post-inoculation, although the titers of ZIKV in seminal fluids were at the limit of detection beginning at 16 days post-inoculation (Figure 4). During the entire 2-week period of infectivity, male-to-female sexual transmission from inoculated AG129 males to uninfected AG129 females was observed (Figure 5), with sexual transmission occurring in 50% of all matings. Viral RNA persisted in semen for weeks beyond the end of the infectious period of semen (Figure 4).

By evaluating the ZIKV shedding kinetics of vasectomized AG129 mice, we showed that testicular cells contribute much of the infectious virus shed in the seminal fluids of mice (Figure 4). In support of these results, we identified ZIKV antigens by immunohistochemistry in the testicular and epididymal epithelia and sloughed germ cells of non-vasectomized males (Figure 3). In comparison, serum and seminal vesicles were devoid of ZIKV by days 7 and 20–25 post-inoculation, respectively. ZIKV RNA was detected in seminal fluids of male AG129 mice for at least 58 days post-inoculation, at which point the study was terminated (Figure 4). This indicates that, for 5 weeks, ZIKV RNA was shed in seminal fluids despite undetectable levels of infectious virus in semen. The source of persistent ZIKV RNA in semen might be the testis/epididymis, which contained detectable ZIKV through at least day 33 (Figure 3); however, vasectomized males also shed viral RNA in the absence of infectious virus (Figure 4). A previous report suggested ZIKV RNA is found in the cellular fraction of semen from humans (Barzon et al., 2016), and another report demonstrated ZIKV antigen in human spermatozoa (Mansuy et al., 2016). Fully characterizing the specific testicular cell types and stages that are permissive to persistent infection requires further studies. The testes/epididymides are an immune-privileged site, and this may contribute to viral persistence in these tissues. However, the persistence of viral RNA in seminal fluids from vasectomized mice presented here suggests that other organs contribute to the prolonged shedding of ZIKV RNA as well.

After ZIKV leaves the testis/epididymis or other organs and enters the seminal fluid, the virus may be neutralized by an adaptive immune response. This could explain the decreased infectivity of ZIKV in semen beginning at day 16 post-inoculation in nonvasectomized mice and at day 9 in vasectomized mice. Whether recrudescent or longer lasting infectious virus can be generated by immunosuppression or co-infection with other infectious agents needs to be explored. Future investigations of viral genetic variation in semen may shed some light on the immune selection and tissue compartmentalization of ZIKV during persistent infection of the testes. The relevance of these studies to human infection with ZIKV is unknown. For example, human spermatogenesis takes 74 days (Heller and Clermont, 1964), whereas the duration of murine spermatogenesis is approximately 35 days (Oakberg, 1957). However, the results described here show that ZIKV RNA can persist in semen without infectious virus, similarly to isolated HIV RNA semen shedding in men on antiretroviral therapy (Sheth et al., 2012), suggesting that an adaptive immune response may be reducing the infectivity of ZIKV shed in semen.

Non-sexual transmission of ZIKV is unlikely to explain the infection of mated females in this study. Infectious ZIKV was not detected in any urine samples collected from 8-week-old male AG129 mice on days 1 through 11 post-inoculation (data not shown). The 21 asymptomatic mated females and 8 unmated females were housed with the 15 infected females and did not seroconvert. However, we cannot rule out a non-sexual method of male-to-female transmission in this study. The very limited study of female-to-male sexual transmission presented here, in which no cases of infection occurred during mating between inoculated AG129 females and uninfected AG129 males, will need to be further expanded in order to assess the risk of female-to-male transmission. Based on a single report of female-to-male sexual transmission in humans, which occurred concurrently with the onset of menses (Davidson et al., 2016), it is possible that lack of a menstrual cycle may preclude mice from being an appropriate model for female-to-male sexual transmission of ZIKV.

Enzootic maintenance of ZIKV is considered to be driven primarily through interactions between non-human primates and mosquitoes (Haddow et al., 1964; Wolfe et al., 2001). However, sexual transmission of ZIKV suggests that horizontal transmission between non-human primates may be an unexplored secondary mechanism for ZIKV maintenance during periods when the principal vectors for transmission—mosquitoes—are not abundant. This study comes on the heels of the recent discovery of the long-lasting persistence of Ebola virus in semen (Soka et al., 2016). The association of both ZIKV and the Ebola virus with sexual transmission potential highlights the need for further studies to assess specific cell tropism and pathophysiological mechanisms that dictate this transmission route for different viruses. The small-animal model described here could be instrumental in resolving some of those questions for ZIKV.

ZIKV is associated with microcephaly and severe brain anomalies in infants (Schuler-Faccini et al., 2016). Therefore, the effects of ZIKV sexual transmission on pregnancy is a major public health concern that needs to be addressed quickly by the scientific community. This mouse model of ZIKV sexual transmission will be an invaluable tool for evaluating the risk of fetal infection via sexual transmission during pregnancy.

## EXPERIMENTAL PROCEDURES

### Inoculation of AG129 Mice

Interferon α/β and -γ receptor knockout AG129 mice were originally obtained from B & K Universal and bred in house. The receptor knockout genotype of the line was confirmed by genetic markers generated from tail snip tissues from ten individuals (Transnetyx). Fourteen 18-week-old male AG129 mice, eleven 13-week-old male AG129 mice, eight 13-week-old female AG129 mice, and eighteen 8-week-old male AG129 mice were inoculated i.p. with 10^3^ PFU of ZIKV strain PRVABC59 (Puerto Rico 2015) (Lanciotti et al., 2016). For 8-week-old mice, three mice were chosen randomly and euthanized on days 1, 3, 5, 7, 9, and 11 post-inoculation, at which point tissues were collected. For all other groups, mice were euthanized when clinical evidence of disease was observed, including recumbency, incoordination, limb weakness, conjunctivitis precluding vision, or difficulty with movement, and tissues were collected. Euthanasia of all animals was performed by deep anesthesia with isoflurane followed by cervical dislocation. Blood was obtained by cardiac puncture while the mouse was under isoflurane anesthesia, and serum was separated by centrifugation of whole blood. Tissues were weighed and homogenized in an equal volume of BA-1 medium using a pestle and clarified by centrifugation. Vaginal washes were collected with 500 µL BA-1 medium using a pipette tip affixed to the end of a 1-mL syringe barrel. Samples were titrated by Vero cell plaque assay, as previously described (Brault et al., 2004), with the modification that the second overlay was added 4 days post-inoculation. The lower limit for this assay was 1.7 log_10_ PFU/mL or 1.4 log_10_ PFU per ejaculate. Statistical significance for differences in viral titers was determined by a t test with a Holm-Sidak correction for multiple comparisons. Survival curves were compared using a log-rank (Mantel-Cox) test. 95% CIs were calculated for proportions, and significance was determined by Fisher’s exact test. Statistical tests were performed in GraphPad Prism. All animal studies were conducted under approved institutional animal care and use committee (IACUC) protocols at the Centers for Disease Control and Prevention.

### Vasectomization of AG129 Mice

Eight 18-week-old AG129 males were anesthetized with 100 mg/kg ketamine-10 mg/kg xylazine. A 1-cm midline incision was made in the caudal ventral abdomen. The testes were partially exteriorized to visualize the vas deferens, the vas deferens was ligated, and a 5-mm section was excised. The incision was closed with absorbable sutures, and the mice were monitored until ambulatory. Meloxicam (10 mg/kg) was provided subcutaneously after recovery from initial anesthesia once daily for 3 days. Vasectomized male AG129 mice were housed individually for 1 week following the procedure, at which point they were mated to CD-1 females to purge any potential spermatozoa from their ejaculatory ducts. After males had been mated to at least one female, they were inoculated i.p. as described earlier.

### Intravaginal Inoculation of AG129 Mice

Female AG129 mice were isoflurane anesthetized and inoculated i.vg. with 10^3^ PFU of ZIKV strain PRVABC59 diluted in 50 µL PBS, using a blunted pipette tip affixed to the end of a 1-mL syringe. Females were inverted for 1 min in order to prevent leakage of inocula. Statistical significance in infection rates was determined by Fisher’s exact test.

### Collection of Seminal Fluids from Male AG129 Mice

Beginning 1 day post-inoculation, 18-week-old male AG129 mice were mated daily to five outbred female CD-1 mice (Charles River Laboratories). Matings were initiated every day by the addition of females to the males’ cage in the evenings and removal the following morning. Successful mating was determined by the presence of a copulatory plug the following morning. Mated female CD-1 mice were immediately isoflurane anesthetized, cardiac bled, and euthanized as described earlier, and the uterus and its contents were collected in 500 µL BA-1 medium. The uterine tissue was homogenized using a pestle and clarified by centrifugation and titrated by Vero cell plaque assay and RNA quantified by real-time RT-PCR (described later). Alternatively, in the second study with 13-week-old male AG129 mice and vasectomized AG129 mice, 500 µL BA-1 medium was used to gavage the uterus contents from both horns. Mated females were replaced each evening to maintain a 5:1 ratio of surrogate female mates to males.

### ZIKV RNA Quantification

Seminal fluids were denatured in 10 mM DTT, and RNA was extracted from denatured samples using the MagMAX Viral RNA Isolation Kit (Ambion). ZIKV RNA was quantified using real-time RT-PCR primers and probe as described previously (Lanciotti et al., 2008). A standard curve was generated by in vitro transcription of a plasmid containing a fragment of ZIKV spanning nucleotides 859–1,278. The detection limit for this assay was 2.0 log_10_ RNA copies per ejaculate.

### Neutralizing Antibody Titrations: PRNT_90_

Surviving mated mice from the sexual transmission studies were tail bled between day 33 and day 36 post-mating (male mice from the female-to-male transmission study) or between day 30 and day 36 post-mating (female mice from the male-to-female transmission study). Serum was heat inactivated at 56°C for 30 min and incubated at a 1:20 dilution with approximately 100 PFU of PRVABC59 for 1 hr at 37°C. Neutralization activity was identified at a 90% plaque reduction threshold.

### Generation of Rabbit Anti-ZIKV Polyclonal Antibody

ZIKV prM and E proteins were cloned into the pAdPL/DEST gateway plasmid (Invitrogen). 293A cells at 85% confluency were transduced with pAd-ZMRprME to generate non-infectious recombinant-adenovirus-vectored ZIKV virus-like particles (VLPs). Anti-ZIKV rabbit polyclonal serum was obtained by three intramuscular immunizations of the VLPs.

### Histology and Immunohistochemistry

Tissues were placed in 10% neutral buffered formalin for 3 days and then stored in 70% ethanol prior to processing for routine paraffin histology. Sections were cut at 4 µm and stained with H&E or by immunohistochemical (IHC) assay for ZIKV antigen prior to evaluation by pathologists (J.M.R and S.R.Z.). The IHC assay was performed using a polymer-based indirect immunoalkaline phosphatase detection system with colorimetric detection of antibody/polymer complex with Fast Red chromogen. The primary antibody was a rabbit polyclonal antibody generated against ZIKV VLPs described earlier and previously shown at the CDC to react with ZIKV antigen in formalin-fixed, paraffin-embedded sections of ZIKV-infected cell cultures and tissues. Appropriate positive and negative controls were performed in parallel.

## Highlights

- Infectious ZIKV is shed in the seminal fluids of infected immunodeficient mice
- ZIKV RNA persists in semen after infectious virus is cleared from semen
- Male-to-female sexual transmission of ZIKV is highly efficient
- Vasectomy significantly reduces the level of infectious ZIKV in semen
